# Case Report: The unrelenting journey—successful resolution of catecholaminergic polymorphic ventricular tachycardia (CPVT) through right cardiac sympathetic denervation in a teenager after left cardiac sympathetic denervation

**DOI:** 10.3389/fcvm.2024.1477359

**Published:** 2024-12-13

**Authors:** Hei-To Leung, Sit-Yee Kwok, Ming Lau, Lucius Kwok-Fai Lee, Sabrina Tsao

**Affiliations:** ^1^ Department of Paediatrics and Adolescent Medicine, Hong Kong Children's Hospital, Kowloon, Hong Kong SAR, China; ^2^ Department of Cardiothoracic Surgery, Queen Mary Hospital, Pok Fu Lam, Hong Kong SAR, China; ^3^ Department of Paediatrics and Adolescent Medicine, The University of Hong Kong, Pok Fu Lam, Hong Kong SAR, China

**Keywords:** catecholaminergic polymorphic ventricular tachycardia (CPVT), right cardiac sympathetic denervation, left cardiac sympathetic denervation, bilateral cardiac sympathectomy, paediatric, case report

## Abstract

**Background:**

Catecholaminergic polymorphic ventricular tachycardia (CPVT) is a rare inherited arrhythmia disorder characterized by ventricular arrhythmia triggered by adrenergic stimulation.

**Case presentation:**

A 9-year-old boy presented with convulsions following physical exertion. Bidirectional ventricular tachycardia (VT) during a treadmill test led to the diagnosis of catecholaminergic polymorphic ventricular tachycardia (CPVT). Genetic testing revealed a pathogenic variant of *RYR2:c.720G>A (p.ArG2401His)*. Nadolol was initially started. However, he experienced aborted VT arrest three years later. Flecainide was thus added as dual therapy and he underwent left cardiac sympathetic denervation (LCSD). Subsequently, a transvenous implantable cardioverter-defibrillator (ICD) was implanted because he still had several episodes of bidirectional VT. Despite a good compliance to medication, the patient still had exercise induced VT episodes with new onset of atrial fibrillation. High dose nadolol was required and amiodarone was added. Despite maximizing the dosage of these three antiarrhythmics, the patient continued to experience multiple episodes of ventricular fibrillation with appropriate ICD shocks and persistent atrial arrhythmias. Right cardiac sympathetic denervation (RCSD) was performed as the last modality of treatment. Patient had a total elimination of VT post bilateral sympathectomy. He remained asymptomatic on follow up. A follow-up treadmill test showed no recurrence of exercise-induced PVCs and VT.

**Conclusion:**

We illustrated the challenges and the complex decision-making process encountered in managing refractory CPVT. In patients unresponsive to conventional therapies, RCSD in additional to LCSD is a safe and effective alternative treatment. A history of LCSD should not preclude physicians from considering RCSD in children with refractory CPVT.

## Introduction

Catecholaminergic polymorphic ventricular tachycardia (CPVT) is an inherited channelopathy characterized by stress-induced bidirectional ventricular tachycardia leading to syncope and sudden cardiac death (1). To reduce the burden of ventricular arrhythmia in CPVT patients, various treatment options have been recommended in the latest European Society of Cardiology guideline in 2022. These include exercise restriction, pharmacological treatments such as β-blockers and flecainide, left cardiac sympathetic denervation (LCSD), and implantable cardioverter-defibrillator (ICD) (1). However, none of these treatments have shown complete efficacy, and in some cases, a combination of these treatments may be necessary to prevent cardiac death. We presented a teenager diagnosed with CPVT who was unresponsive to all of the aforementioned treatment. Finally, he underwent thoracoscopic right cardiac sympathetic denervation (RCSD), resulting in complete suppression of ventricular tachycardia (VT).

## Case report

**Figure 1:**
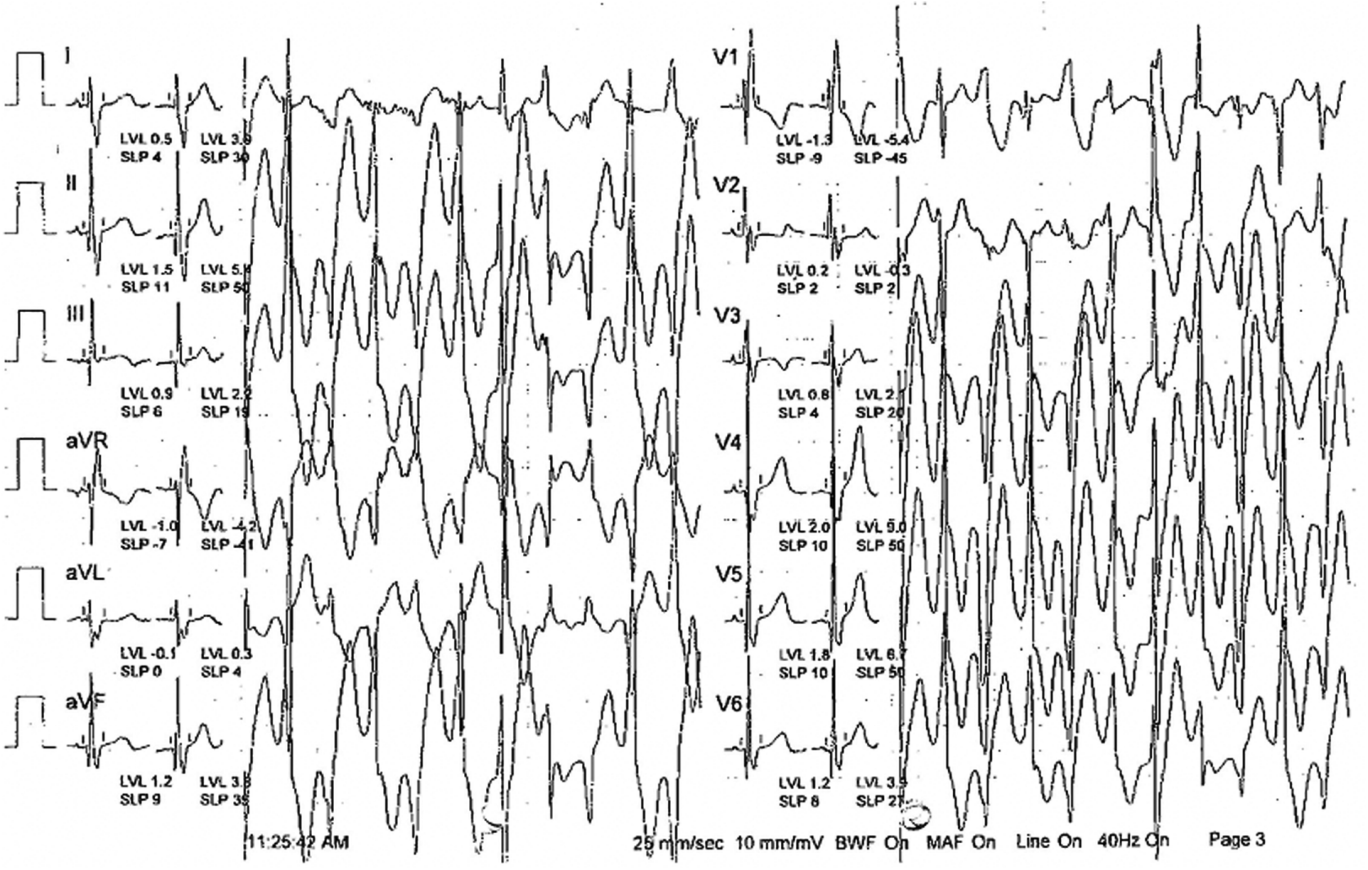
Stress exercise test revealed bidirectional ventricular tachycardia.

A 9-year-old Pakistani boy had been diagnosed with epilepsy and experienced repeated convulsions since the age of 4. Physical examination was unremarkable. However, subsequent convulsions occurring after physical exertion raised suspicion of arrhythmia syndrome. A stress exercise test revealed bidirectional ventricular tachycardia, leading to a diagnosis of CPVT (Figure 1). A genetic test confirmed the presence of a *de novo* heterozygous likely pathogenic RYR2 variant *(RYR2:c.14861C>G; p.Ala4954Gly)*. He was started on non-selective beta blocker i.e., nadolol and experienced no syncope or convulsions thereafter. He remained symptom-free for 4 years. However, at the age of 13, he had an episode of aborted cardiac arrest, prompting the optimization of nadolol dosage to 40 mg daily (1.3 mg/kg/day) and the addition of flecainide 100 mg twice daily (6.6 mg/kg/day). LCSD was performed to enhance patient protection. The follow-up treadmill test was negative.

**Figure 2:**
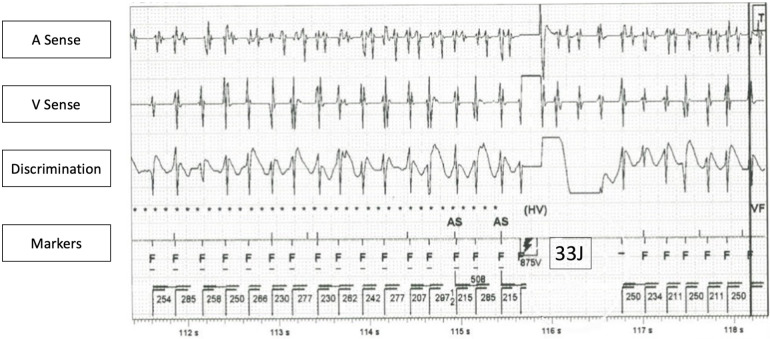
ICD interrogation of a VT episode, which failed to be terminated by ICD shock. (Remarks: Concurrent atrial fibrillation also noted in atrial channel, also failed to terminate by ICD shock).

Two years following LSCD, the patient experienced syncope while playing boxing, and electrocardiogram (ECG) on ambulance revealed bidirectional ventricular tachycardia (VT), leading to the implantation of a transvenous dual-chamber implantable cardioverter-defibrillator (ICD) as secondary prevention. The ICD programming was set as follows: VT-1 zone was set at 200–230 beat per min (bpm), with a detection interval of 100 s. Six antitachycardia pacing (ATP) therapies were programmed before shock therapy. VT-2 zone was set at 230–260 bpm with the same detection interval. Three ATP therapies would been given before a shock is delivered. Ventricular fibrillation (VF) zone was set at heart rate exceeding 260 bpm, in which an ATP therapy was administered during charging. The rationale behind the VT-1 and VT-2 detection settings and an extended detection period, was to address clinical VT while minimizing the necessity for shocks that could trigger a VT storm. Despite this, patient still received multiple appropriate ICD shocks. ICD interrogation showed multiple episodes of polymorphic VT although the patient remained asymptomatic (Figure 2). ATP and ICD shocks were all ineffective in terminating these VT but they all reverted back to sinus rhythm spontaneously. Defibrillation threshold test was normal. The ICD was reprogrammed as follows to enhance our detection of the slow clinical VT: VT-1 monitoring was set at 176 bpm. VT-2 zone was set at 230–260 bpm, in which three ATP therapies would been given before a shock is delivered. Ventricular fibrillation (VF) zone was set at heart rate exceeding 260 bpm, in which one ATP therapy would be administered before shock delivery. Two years later, during ICD interrogation, the patient was found to have atrial fibrillation with rapid conduction, and the dosage of nadolol and flecainide were increased to 120 mg twice daily (4.5 mg/kg/day) and 150 mg twice daily (5.6 mg/kg/day) respectively. However, atrial fibrillation persisted. Amiodarone 200 mg daily (3.8 mg/kg/day) was added for rhythm control and subsequent treadmill test showed no exercise induced ventricular arrhythmia.

**Figure 3:**
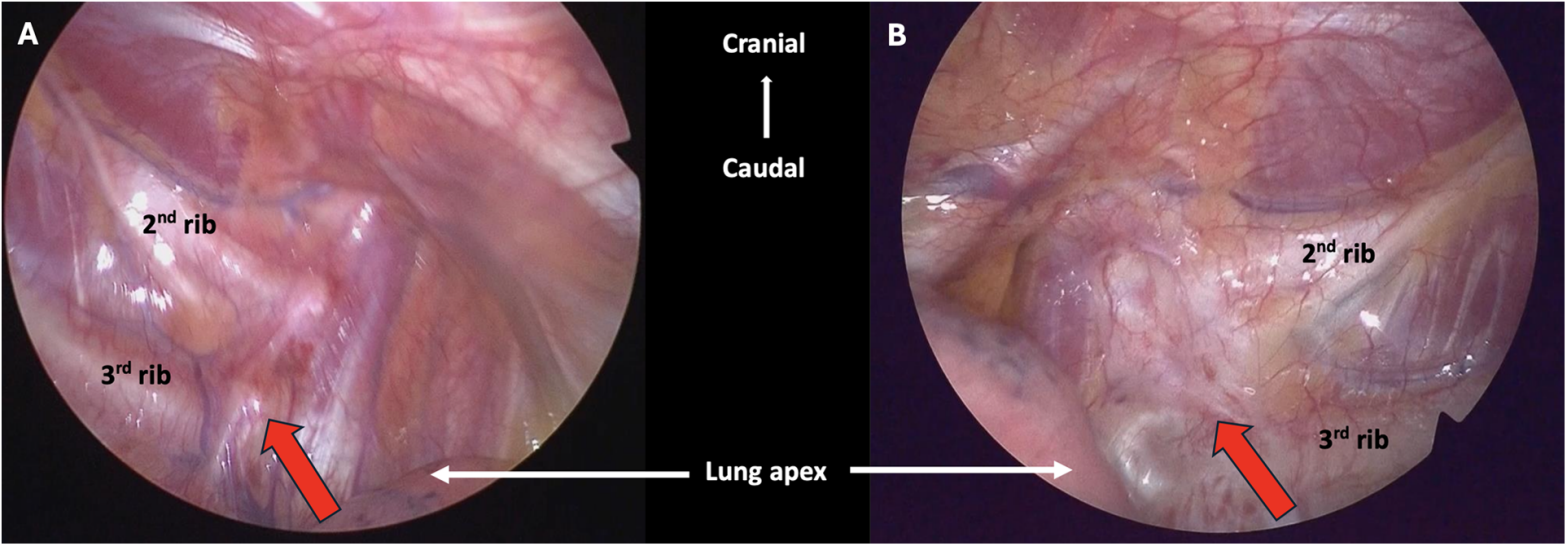
Intraoperative findings during bilateral cardiac sympathectomy. **(A)** The red arrow pointing to the right sympathetic trunk. **(B)** The red arrow pointing to the fibrotic left sympathetic trunk (status post LCSD).

Despite maximizing medical therapy with three anti-arrhythmic medications, he experienced multiple episodes of VF with appropriate ICD shocks and ongoing atrial arrhythmias. The episodes of VF were triggered either by emotions, dumbbell exercise, or playing computer games. The patient expressed dissatisfaction with adhering to exercise restrictions and taking numerous medications. Therefore, thoracoscopic exploration of left cardiac sympathetic stellate ganglion and right cardiac sympathetic denervation (RCSD) was performed. Intraoperatively, fibrous tissue was found over the left paraspinal region at the T1–T4 level, and no residual neural tissue was seen (Figure 3). The parietal pleura along the left second rib was cauterized. In view of satisfactory left-side denervation, we proceeded to the right-sided operation. Inferior stellate ganglionectomy, T2–T4 sympathectomy, and Kuntz's nerve ablation were performed (Supplementary video). Histopathological examination confirmed the presence of nerve bundles containing ganglion cells on the right side. The procedure was uneventful, and he was discharged on the day after the operation. Patient remained asymptomatic on follow up. He did not receive any ICD shocks and there was no more atrial and ventricular tachycardia detected. A subsequent treadmill test conducted three months after the sympathectomy showed no exercise induced PVCs and VT. The heart rate response was blunted, with a maximal heart rate of 76 beats per min achieved (Table 1). He remained asymptomatic and his antiarrhythmic medications were gradually tapered down. He was on nadolol 80 mg daily and flecainide 100 mg twice daily on 6-months follow-up.

**Table 1:** Summary of treadmill test results before and after LCSD and RCSD.

	Before LCSD	After LCSD	Before RCSD	After RCSD
Treadmill test results				
Rest HR (bpm)	Sinus rhythm	Sinus rhythm	Sinus rhythm	Atrial paced rhythm
	56 bpm	60 bpm	73 bpm	60 bpm
Peak HR (bpm)	VT	Sinus rhythm	Sinus rhythm	Sinus rhythm
	179 bpm	139 bpm	96 bpm	76 bpm
Induced arrhythmia	Bidirectional VT	PVCs only, no VT	Occasional PVC at rest, No VT	No PVCs
Threshold at onset	120 bpm	2 monomorphic PVCs noted at recovery	None during exercise	No PVC VT
Medications	Nadolol 40 mg daily	Nadolol 40 mg daily	Nadolol 120 mg twice daily	Nadolol 120 mg twice daily
		Flecainide 100 mg twice daily	Flecainide 200 mg/150 mg twice daily Amiodarone 200 mg daily	Flecainide 100 mg twice daily Amiodarone 100 mg daily
Dual chamber ICD programming and interrogation results				
ICD program	N/A	VT-1 zone: 200–230 bpm, ATP × 6 → shock	VT-1 monitoring: >176 bpm	VT-1 monitoring > 176 bpm
		VT-2 zone: 230–260 bpm, ATP × 3 → Shock	VT-2 zone: 230–260 bpm, ATP × 3 → Shock	VT-2: 230–260 bpm, ATP × 3 → Shock
		VF zone: >260 bpm, ATP during charging, followed by shock	VF: >260 bpm, ATP × 1 → Shock	VF zone: >260 bpm, ATP × 1 → Shock
Percentages of atrial and ventricular pacing		Atrial pacing 60%	Atrial pacing 81%	Atrial pacing 70%
		Ventricular pacing <1%	Ventricular pacing <1%	Ventricular pacing <1%

ATP, anti-tachycardia pacing; bpm, beat per minute; ICD, implantable cardioverter-defibrillator; LSCD, Left Cardiac Sympathetic Denervation; RSCD, Right Cardiac Sympathetic Denervation; VF, ventricular fibrillation; VT, ventricular tachycardia.

## Discussion

CPVT is a rare inherited arrhythmia characterized by the occurrence of bidirectional polymorphic ventricular tachycardia (VT) triggered by the presence of adrenergic stress. The primary approach in managing arrhythmic events in CPVT has traditionally involved the use of β-blockers, which target the underlying catecholaminergic mechanism of the arrhythmia (1). Among the β-blockers, non-selective β-blockers like nadolol and propranolol have been associated with a significant risk reduction of arrhythmic events in children compared to β1-selective β-blockers (2). Regarding the nadolol dosage for CPVT, the recommended daily dose was 2 mg/kg/day, with a median dosage of 1.1 (0.8–1.6) mg/kg/day reported in an international cohort (2). In our case, we attempted to administer nadolol at a notably high dosage of up to 5 mg/kg/day, inspired by its previous use in infants with supraventricular tachycardia (3). Despite this high dosage in our patient, there was no additional benefit observed, and as far as our knowledge extends, there is no supportive evidence for the efficacy of nadolol doses exceeding 2 mg/kg/day in CPVT patients.

In recent studies, flecainide has also demonstrated efficacy in reducing arrhythmic events (4). The mechanism of action of flecainide, whether through modulation of sodium channel-mediated intracellular calcium dynamics or inhibition of the cardiac ryanodine receptor (*RYR2*), remains a subject of ongoing debate (5). Unfortunately, although these medications show promising effects, none of them offer complete protection, as 9% of patients on flecainide and 13% of patients on β-blockers still experience arrhythmic events (2, 4, 6). In patients refractory to medical treatment, ICD was recommended despite its variable efficacy, which depend on the mechanism of ventricular arrhythmia, tachycardia cycle length and the presence of supraventricular arrhythmia (1, 7). It is important to note that the use of ICD is intended for “secondary prevention” of ventricular arrhythmia only, although it's essential to consider that repetitive shocks could precipitate an arrhythmic storm. For this young gentleman, albeit optimizing all the aforementioned treatments, he still experienced frequent breakthrough VT. This prompted us to consider alternative options, ultimately leading to the decision to perform cardiac sympathectomy.

Cardiac sympathectomy, recognized as a last resort for medically refractory CPVT, involves removal of the lower third to half of the stellate ganglion (T1), together with the thoracic ganglia from T2 to T4 (1, 8). Initially, Moss and McDonald described the use of LCSD in long QT syndrome (LQTS), based on the belief that increased sympathetic activity on the left side contributes to LQTS (9). This sparked interest in the potential antiarrhythmic benefits of LCSD, leading to its application in other life-threatening arrhythmias, including CPVT (8). In recent decades, our understanding of the role of cardiac sympathetic denervation in ventricular arrhythmia has advanced and found that the molecular mechanisms in the final anti-arrhythmic effect was boarder than expected. Specifically, research has revealed the significant involvement of the stellate ganglion in the conduction system and heart contraction, predominantly through ß1-adrenergic receptors and noradrenaline activity. In addition, the stellate ganglion also releases neuropeptide Y (NPY) to modulate sympathetic activity not only by inhibiting acetylcholine release from parasympathetic neurons, but also through NPY receptors present on cardiomyocytes and coronary arteries (10). Consequently, the removal of the stellate ganglion during sympathectomy not only mitigates the decrease in norepinephrine release and counteracts the effects of neuropeptide Y, but also the reduction in sympathetic stimulation. This reduction leads to a decrease in the overall dispersion of repolarization, thereby exerting its antiarrhythmic effects (11, 12). While LSCD has shown effectiveness in reducing arrhythmia burden in CPVT, its recurrence is possible. In the most extensive study on LSCD in CPVT conducted by Ferrari et al., although the findings showed promising outcomes with a reduction in significant cardiac events, 32% of patients still encountered major cardiac events following LSCD (13). Another case series by Hofferbecrth et al., only four out of nine CPVT patients remained free of ICD shocks after LSCD, and similar ineffectiveness was observed in our patient two years post-procedure, leading to the need for ICD implantation (14). Despite being aware of the potential for LSCD failure, upon reviewing the 2017 American Heart Association/American College of Cardiology (ACC)/Heart Rhythm Society and 2022 European Society of Cardiology guidelines on management of ventricular arrhythmias, it is evident that both guidelines endorse LCSD as a last-resort option but do not offer recommendations on handling cases where LCSD has proven ineffective (1, 15). Our patients underwent thorough medical and surgical interventions as per the recommendations in these guidelines; however, he continues to experience breakthrough arrhythmias. Our experience highlights the potential effectiveness of RSCD for patients with resistant CPVT after LCSD attempts.

The inefficacy of LCSD has been theorized to stem from the hypertrophy of the right cardiac sympathetic chain, which can reinnervate areas previously serviced by the surgically excised left ganglia (16). Additionally, it has been described in patients suffering from heart failure and structural heart disease may experience a restructuring of postganglionic sympathetic neurons in the bilateral stellate ganglia due to pronounced abnormal afferent signaling, potentially resulting in severe arrhythmias (17, 18). Considering these factors, bilateral cardiac sympathectomy emerges as a reasonable alternative for patients with medically refractory ventricular arrhythmia. This approach has been supported by a study conducted by Vaseghi et al., which demonstrated a notable decrease in ICD shocks in adults with medically resistant ventricular arrhythmia who underwent bilateral cardiac sympathectomy compared to those who had LSCD. Given that most patients in this research had underlying cardiomyopathy, there was a compelling argument to initiate treatment directly with bilateral cardiac sympathetic denervation rather than LCSD (19). However, this does not diminish in any way the additional value of right cardiac sympathetic denervation in very selected cases of CPVT patients with LCSD refractory arrhythmias, albeit with limited supporting data from a handful of case reports that highlight its safety and efficacy in adult populations (20, 21). In a study conducted by Ertugrul et al., positive outcomes were observed in a group of fourteen children aged 8–19 who underwent bilateral cardiac sympathectomy. Among these children, six patients had CPVT. The study findings indicated that there were no instances of ventricular arrhythmia recurrence in the children with CPVT during the follow-up period (22).

It is important to mention that bilateral cardiac sympathectomy was rarely performed on younger children due to clinician concerns about potential complications like Horner's syndrome, harlequin facial flushing, dry skin, significant bradycardia, or neuropathic pain. Generally, sympathectomy has shown to be a safe procedure in children and pain are usually short-lasting (22). Specifically, clinicians might be apprehensive about the possibility that bilateral cardiac sympathectomy could reduce chronotropic competence and escalate the necessity for atrial pacing (23). On top of that, prior literature has highlighted that the primary innervation of the sinoatrial node originates from cardiac postganglionic sympathetic nerve terminals in the right stellate ganglion (17). Therefore, additional limitation to heart rate increase provided by RCSD on top of LCSD, could explain the effectiveness of RCSD or bilateral cardiac sympathectomy in the very few cases with severe and recurring malignant arrhythmias after LCSD.

Our case study further exemplified the potential benefits of RCSD in CPVT patients underwent LCSD, as it showcased how this procedure can help avoid complications associated with implantable cardioverter-defibrillators (ICDs) and mitigate the side effects of high-dose antiarrhythmic drugs. More importantly, our case also demonstrated that RSCD is a safe procedure, even for children with a history of LSCD, as it does not pose a higher risk in such cases.

## Conclusion

For refractory CPVT patients with treatment failure using conventional therapies, RCSD can be considered as a safe and effective alternative treatment. A history of previous LCSD should not deter clinicians from considering RCSD as a potential therapeutic approach for these patients.

## Data Availability

The original contributions presented in the study are included in the article/Supplementary Material, further inquiries can be directed to the corresponding author.
